# Complete Mitogenome and Phylogenetic Analyses of *Galerita orientalis* Schmidt-Goebel, 1846 (Insecta: Coleoptera: Carabidae: Galeritini)

**DOI:** 10.3390/genes13122199

**Published:** 2022-11-23

**Authors:** Yu Bai, Kang Yang, Lin Ye, Xuyuan Gao

**Affiliations:** 1 College of Mathematics & Information Science, Guiyang University, Guiyang 550005, China; 2 College of Biology and Environmental Engineering, Guiyang University, Guiyang 550005, China; 3 Guangxi Key Laboratory of Biology for Crop Diseases and Insect Pests, Institute of Plant Protection, Guangxi Academy of Agricultural Sciences, Nanning 530007, China; 4 Key Laboratory of Green Prevention and Control on Fruits and Vegetables in South China Ministry of Agriculture and Rural Affairs, Institute of Plant Protection, Guangxi Academy of Agricultural Sciences, Nanning 530007, China

**Keywords:** *Galerita orientalis*, mitochondrial genome, phylogenetic analysis

## Abstract

The genus *Galerita* Fabricius, 1801 belongs to the tribe Galeritini of the family Carabidae. In this study, the complete mitochondrial genome (GenBank: ON920164.1) of *G*. *orientalis* is newly sequenced, annotated, characterized, and composed of 37 typical genes, and one control region. Mitogenome is a circular DNA molecule of 16,137 bp with a 78.79% AT content. All 13 protein-coding genes are initiated using a typical ATN (Met) as the start codon, except for *nad1*, which has a TTG as the start codon, and are terminated using a typical TAN stop codon. Twenty-two tRNAs could fold into a typical cloverleaf structure, including trnS1-GCU, which lacks the DHU stem observed in other mitogenomes of the subfamily Harpalinae. Both rrnS and rrnL contain many helices. A conserved poly-T stretch (19 bp) and seven tandem repeats are observed in the control region, and a phylogenetic analysis indicated that the genus *Galerita* is an independent lineage. The complete mitogenome of *G*. *orientalis* will contribute to further studies on the molecular basis of the classification and phylogeny of Harpalinae, and even Carabidae.

## 1. Introduction

Carabidae is the largest family in the order Coleoptera, comprising more than 32,000 species worldwide [1,2], and is known as carabid beetles or ground beetles [1,3]. Harpalinae is the largest group of Carabidae and contains more than 19,000 species [1,4]. These beetles live in diverse habitats, have diverse morphological forms, and exhibit a variety of unusual lifestyles [5]. Therefore, the study of phylogeny is critical for understanding the evolution and diversity of tribes, genera, and species within the Harpalinae [1,5]. The monophyly of Harpalinae is based on morphological characteristics, chemical defensive secretions, chromosome number in males, and molecular sequence data obtained from the 18S rDNA gene [1,5]. However, the boundaries of these character states do not exactly match Harpalinae [1,5]. The nominotypical subgenus of the pantropical genus *Galerita* Fabricius, 1801 is represented in all zoogeographic regions, except Australian [6,7,8,9], and includes more than 110 species [10], which belong to the tribe Galeritini of the subfamily Harpalinae. The taxonomy of the tribe Galeritini has been developed based on adult and larvae features [6,7,8,9,10,11]. Based on the DNA-sequence datasets obtained from nuclear genes (the 28S rDNA and wingless nuclear protein-coding genes), higher-level phylogenetic relationships within Harpalinae are investigated [1,5,12,13], including the tribe Galeritini. *Galerita* is a sister group to ctenodactylines in the Zuphiitae clade, based on the 28S rDNA gene and wingless data [13].

**Figure 1:**
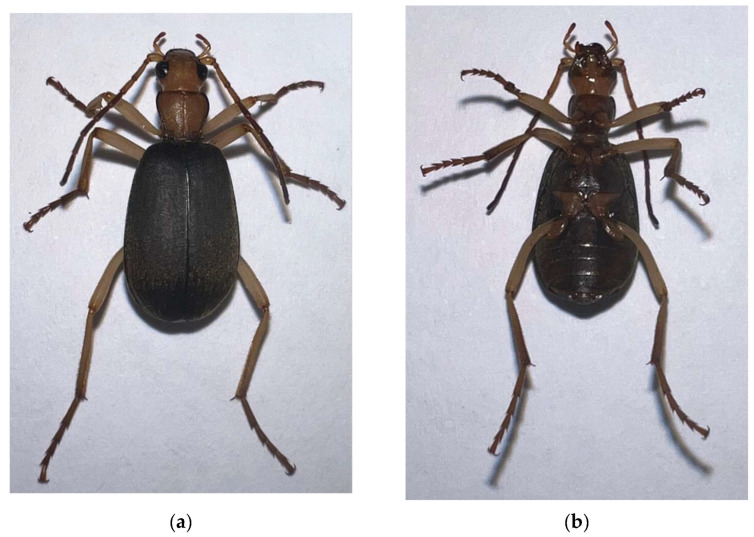
Species reference image of *Galerita orientalis* Schmidt-Göbel, 1846. (**a**) The dorsal view of *G*. *orientalis*; (**b**) the ventral view of *G*. *orientalis*.

*Galerita orientalis* Schmidt-Göbel, 1846 (Figure 1) is widely distributed in continental Asia, Japan, and the Greater and Lesser Sunda Islands [9], and was revised by Reichardt (1965) [8,9]. However, our knowledge remains incomplete, with limited genetic and mitogenomic information from *G*. *orientalis*. As of August 2022, only three partial coding sequences of *cox1* from *G*. *orientalis* had been published in GenBank. Insect mitochondrial genomes (mitogenomes) are closed, circular, double-stranded DNA molecules with lengths ranging from 15 to 19 kb that contain 37 typical genes, including 13 protein-coding genes (PCGs), 2 rRNAs, 22 tRNAs, and a control region (CR). Mitogenomes play an important role in the molecular phylogeny of insects [14]. Next-generation sequencing (NGS) is an important and effective strategy for mitogenome assembly and the phylogenetic analysis of Harpalinae [3,15,16,17].

In the present study, we sequence and characterize the *G*. *orientalis* mitogenome using NGS. Furthermore, we construct phylogenetic trees based on the mitogenomes of 30 species of the family Carabidae and two outgroup species, which will contribute to the research on its phylogenetic position in the family Harpalinae. These results will be useful to reconstruct the phylogenetic relationships within Carabidae in the future.

## 2. Materials and Methods

### 2.1. Animal Materials, DNA Extraction, and Illumina Sequencing

Samples of adult *G*. *orientalis* were collected from Jingziguan Town (E 111.026°, N 33.244°), Xichuan County, Nanyang City, Henan Province, China, on 9 June 2022, the genomic DNA (gDNA) of which was extracted using the Qiagen DNeasy Blood and Tissue Extraction kit (Qiagen, Germantown, MD, USA). The purity and concentration of the obtained gDNA were tested using a NanoPhotometer^®^ spectrophotometer (Implen, Calabasas, CA, USA) and a Qubit^®^ 2.0 fluorometer (Life Technologies, Carlsbad, CA, USA), respectively [18,19]. Sequencing libraries for the quality-checked gDNA were generated using a TrueLib DNA Library Rapid Prep Kit for Illumina sequencing (Illumina, Inc., San Diego, CA, USA) [18,19]. The libraries were subjected to size-distribution analysis using an Agilent 2100 bioanalyzer (Agilent Technologies, Inc., Santa Clara, CA, USA), followed by a real-time PCR quantitative test [18,19]. The successfully generated libraries were sequenced using an Illumina NovaSeq 6000 platform (Illumina, Inc., San Diego, CA, USA) [18,19], and 150 bp paired-end reads with a 300 bp insert library were generated.

### 2.2. DNA Data Cleaning and Mitogenome Assembly

The obtained raw reads were filtered to obtain clean reads using fastp version 0.23.2 (https://github.com/OpenGene/fastp (accessed on 23 October 2022)) [20]. The quality control (QC) standards of the reads obtained from the DNA were as follows:

(1) Trimming adapter sequences with >6 bases;
(2) Removing reads with >0 unidentified nucleotides (Ns);
(3) Removing reads with >20% bases with Phred quality < Q30;
(4) Removing reads with <150 bases.

The mitogenome of *G*. *orientalis* was assembled de novo from high-quality cleaned reads using NOVOPlasty v4.3.1 (https://github.com/ndierckx/NOVOPlasty (accessed on 23 October 2022)) [21] with default parameters and the *G*. *orientalis* voucher NSMK-IN-160200486 *cox1* gene (GenBank: OL663076.1) [16] as a seed sequence.

### 2.3. Mitogenome Annotation and Analysis

The AT-skew [(A − T)/(A + T)] and GC-skew [(G − C)/(G + C)] of the sequence were estimated to investigate the nucleotide composition bias, using Perna and Kocher’s formula [22]. The *G*. *orientalis* mitogenome was initially annotated using GeSeq version 2.03 (https://chlorobox.mpimp-golm.mpg.de/geseq.html (accessed on 23 October 2022)) [23], using the third-party software tRNAscan-SE v2.0.7 [24], ARWEN v1.2.3 [25], BLAT v36×7 [26], with the mitogenome of *Mastax latefasciata* (ON674050.1) as a reference. Using the mitogenomes of *M*. *latefasciata* as references, the start and stop codons of PCGs were manually corrected. The order and orientation of the genes were determined and drawn using the CGView Web server (https://proksee.ca/ (accessed on 23 October 2022)) [27]. Relative synonymous codon usage (RSCU) was analyzed using MEGA v11.0.13 [28]. The secondary structures of tRNAs were analyzed and visualized using forna [29,30]. The secondary structures of rRNAs and CR were inferred and visualized using RNAfold Web server (http://rna.tbi.univie.ac.at/cgi-bin/RNAWebSuite/RNAfold.cgi (accessed on 23 October 2022)) [31,32], with minimum free energy (MFE) and partition functions [33]. Tandem repeats in the control region (CR) were predicted using the Tandem Repeats Finder program version 4.09 [34] with parameters = 2 7 7 80 10 50 500 -f -d m.

### 2.4. Phylogenetic Analysis

In this study, the phylogenetic analysis included sequences from mitogenomes of 30 Carabidae species and 2 outgroup species (*Lepisma saccharina* and *Corydidarum magnifica*) (Table 1). The concatenated sequences of 13 protein-coding genes from mitogenomes were used to reconstruct the phylogenetic relationships of Carabidae using PhyloSuite version 1.2.2 [35] with MAFFT version 7 [36], MACSE version 2.03 [37], Gblocks 0.91b [38], ModelFinder [39], MrBayes version 3.2.7 [40], and IQ-TREE 1.6.12 [41]. Nucleotide sequences of 13 PCGs were aligned using MAFFT version 7 with the default parameters, and MACSE version 2.03 with default parameters. Ambiguously aligned fragments of the alignments from the 13 PCGs were removed using Gblocks 0.91b [38] with default parameters. The nucleotide sequences were used to construct phylogenetic trees using two methods: Bayesian inference (BI) using MrBayes 3.2.0,7 and maximum likelihood (ML) using IQ-TREE 1.6.12. According to the Bayesian information criterion (BIC) scores, a GTR + F [state frequencies, fixed (empirical)] + I [proportion of invariable sites, uniformly distributed on the interval (0.00, 1.00)] + G4 (γ-distributed rate variation, four categories) model was selected as the best-fit partition model (edge-unlinked) for the BI of nucleotide sequences using PhyloSuite version 1.2.2 with ModelFinder. For the IQ-tree, owing to the selection of the partition mode, the best-fit partition model was automatically calculated before the phylogenetic trees were constructed. In the BI analysis, 2 runs of 2,000,000 generations were conducted for each matrix, and the initial 25% was discarded as burn-in, which had the same topology with an average standard deviation of split frequencies of 0.008349 (<0.01). In the ML analysis, node support values were assessed using 5000 bootstrap resampling replicates. The resulting phylogenetic trees were visualized using an interactive tree of life (iTOL) (https://itol.embl.de/ (accessed on 23 October 2022)) [42].

## 3. Results and Discussion

### 3.1. Sequencing, Quality Control, and Mitogenome Organization and Base Composition of G. orientalis

Approximately 43.38 Gb of high-quality, clean reads were obtained using the fastp software [6] from approximately 51.89 Gb of raw reads of a 300 bp insert library, using the Illumina NovaSeq 6000 platform for the *G. orientalis* mitogenome assembly. The Q20, Q30, and GC contents of the clean reads were 98.37%, 93.88%, and 35.88%, respectively (Table 2).

These high-quality clean short reads (0.39% reads from the mitogenome) defined the mitogenome of *G*. *orientalis* (ON920164.1) with 100% coverage at a high-average-reads depth (10,425 times), which consisted of a typical, single, circular DNA molecule 16,137 bp in length. The length of this mitogenome was between 16,027 bp for *Ha*. *discrepans* and 17,701 bp for *Ab*. *parallelepipedus* in the subfamily Harpalinae (Table 1).

The mitogenomes of *Cr*. *nobilis* and *He*. *terminalis* were sequenced using the Roche/454 sequencing platform [49]. The mitogenomes of *Ab*. *parallelepipedus*, *S*. *pumicatus*, *P*. *niger*, *P*. *madidus*, *Ha*. *sinicus*, *Ha*. *pensylvanicus*, and *Am*. *aulica* were sequenced on an Illumina sequencer [3,15,16,17]. Therefore, the complete Harpalinae mitochondrial genome could be assembled using NGS alone.

The mitogenome of *G*. *orientalis* contained 40.85% A, 37.94% T, 8.46% G, and 12.75% C, which showed an obvious AT bias with 78.79% AT content. The AT content of the *G*. *orientalis* mitogenome was slightly lower than those of *Ha. sinicus* [16], *Am*. *communis* [17], *S. pumicatus* [17], and *Ha. pensylvanicus* [17]. The AT- and GC-skews of the major strand of the *G*. *orientalis* mitogenome were 0.037 and −0.202, respectively, indicating a major strand compositional bias characterized by a slight excess of A over T nucleotides, and a strong excess of C over G nucleotides. Bias is generally observed in the mitogenomes of members of the subfamily Harpalinae, including *Am. aulica* [3] and *Ha. pensylvanicus* [17].

**Figure 2:**
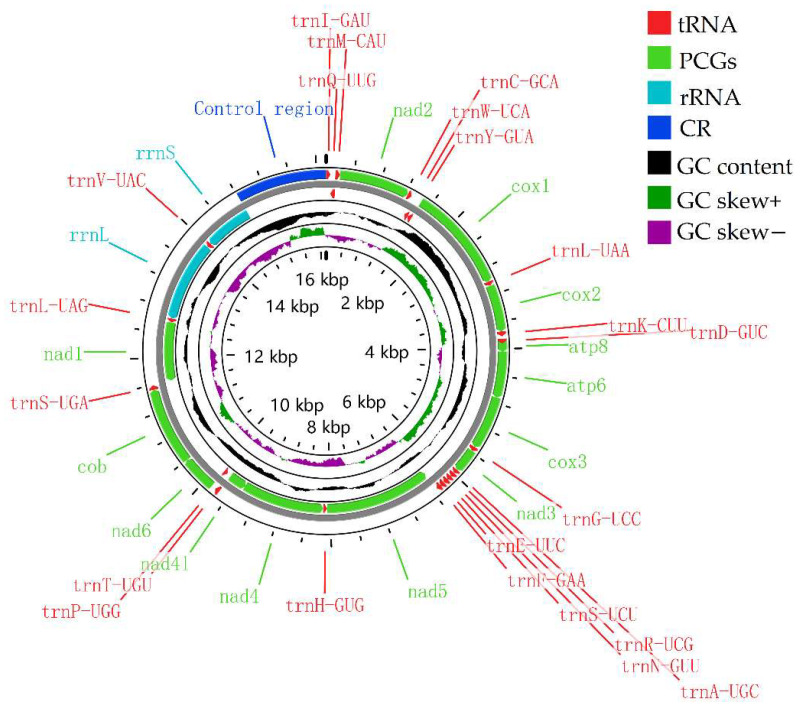
Mitogenome-pattern map of *G. orientalis*. The first circle shows the gene map (PCGs, rRNAs, tRNAs, and control region). Arrows in the second and third circles indicate the direction of gene transcription. The fourth circle shows the GC content and the fifth shows the GC-skew values. GC content and GC skew are plotted as the deviation from the average value of the entire sequence.

The *G*. *orientalis* mitogenome comprises 13 PCGs, 22 tRNA, 2 rRNA, and 1 CR (Figure 2), of which the order and orientation of the genes are the same as those in the mitogenomes of the subfamily Harpalinae [3,15,16,17,49]: 23 genes (9 PCGs and 14 tRNAs) on the majority strand (J-strand), and 4 PCGs, 8 tRNAs, and 2 rRNAs on the minority strand (N-strand) (Figure 2 and Table 3). Specifically, the mitogenome contains 12 overlapping genes (a total of 42 bp), ranging from 1 to 8 bp, with the longest region located between trnY-GUA and *cox1* in the *Ha. sinicus* mitogenome [16] (Table 3). Most of the gene-overlap regions appeared between tRNA and PCGs. Intergenic spacers have 10 regions (a total of 45 bp), ranging from 1 to 16 bp, with the longest region located between 20 trnS-UGA and *nad1* in the *Ha. sinicus* mitogenome [16] (Table 3).

### 3.2. Protein-Coding Genes

A total of 9 of the 13 PCGs were encoded on the majority strand (*cox1*, *cox2*, *cox3*, *atp6*, *atp8*, *nad2*, *nad3*, *nad6*, and *cob*), and 4 on the minority strand (*nad1*, *nad4*, *nad4l*, and *nad5*) (Figure 2 and Table 3). All 13 PCGs had a typical ATN (Met) start codon, except for *nad1* (TTG as start codon): only 1 PCG (*nad2*) initiated with an ATA start codon, 5 PCGs (*cox1*, *nad3*, *nad5*, *nad4l*, and *nad6*) initiated with an ATT start codon, 5 PCGs (*cox2*, *atp6*, *cox3*, *nad4*, and *cob*) initiated with an ATG start codon, and only 1 PCG (*atp8*) initiated with an ATC start codon. The start codons in the *G*. *orientalis* mitogenome were consistent with those in the *Ha. sinicus* mitogenome. In the mitogenomes of the subfamily Harpalinae, CGA and TAT are used as start codons [3,17]. All 13 PCGs contained a typical TAN stop codon, 3 PCGs (*nad3*, *cob*, and *nad1*) terminated with a TAG stop codon, 6 PCGs (*nad2*, *atp8*, *atp6*, *cox3*, *nad4l*, and *nad6*) ended with a TAA stop codon, and 4 PCGs (*cox1*, *cox2*, *nad5*, and *nad4*) terminated with an incomplete stop codon (T), consisting of a codon that was completed by the addition of A nucleotides at the 3′ end of the encoded mRNA. Most of the stop codons of the genes in the *G*. *orientalis* mitogenome were identical to those in the *Ha. sinicus* and *Am. aulica* mitogenomes. Other types of stop codons are present in the mitogenomes of the subfamily Harpalinae, such as incomplete stop codons A, CTA, and TTA [16,17]. The diversity of the start and stop codons reflects the evolutionary diversity of species and makes it difficult to determine the start and stop positions of PCGs.

**Figure 3:**
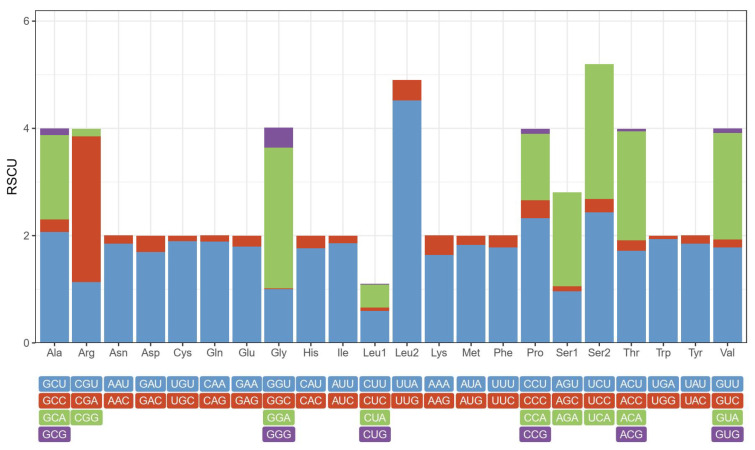
Relative synonymous codon usage (RSCU) of *G. orientalis* mitogenome.

The results of the relative synonymous codon usage (RSCU) analysis for the 13 PCGs, comprising 3714 codons excluding the start and stop codons, showed codon usage bias in the *G*. *orientalis* mitogenome (Figure 3 and Supplementary Table S1). Among the amino acids (Supplementary Table S1), Leu was the predominant type (576), followed by Ile (381) and Phe (363). Among the codon usage counts, UUA (434) for Leu was dominant, followed by AUU (356) for Ile and UUU (323) for Phe. Thirteen PCGs had the biased usage of the A and T nucleotides (Supplementary Table S2).

### 3.3. Transfer and Ribosomal RNA Genes

**Figure 4:**
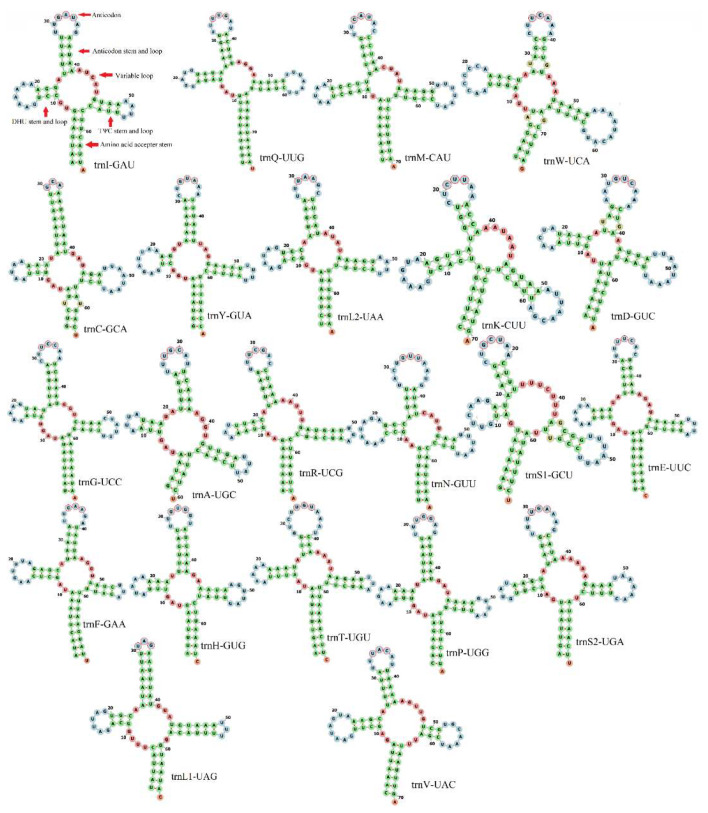
Secondary structure of 22 tRNAs of *G. orientalis* mitogenome. The mismatched base pairs are presented in yellow; the matched base pairs are presented in green; the bases in the loop are presented in blue; and the nucleotide outlines of the anticodon are presented in red.

The traditional 22 tRNA genes were interspersed among the PCGs. Fourteen of the 22 tRNAs were in the majority strand, and eight were in the minority strand (Figure 2 and Table 3). The lengths of the 22 tRNAs ranged from 61 bp (trnA-UGC) to 75 bp (trnQ-UUG and trnW-UCA) (Table 3 and Supplementary Table S2), which had a typical cloverleaf secondary structure (Figure 4). Most of the secondary structures of tRNAs in the *G*. *orientalis* mitogenome were consistent with those in the mitogenomes of *Ha. sinicus* and *Ha. pensylvanicus*. In the mitogenomes of the subfamily Harpalinae, trnS1-GCU lacked a DHU stem [3,16,17], which was replaced by a simple loop [3,16,17]. However, the trnS1-GCU of the *G*. *orientalis* mitogenome had a 2 bp DHU (Figure 4). The diversity of the secondary structures of tRNAs reflects the evolutionary diversity of the species. The length of the anticodon stems of the tRNAs ranged from 3 bp (trnT-UGU) to 8 bp (trnC-GCA) (Figure 4). The length of the DHU stem ranged from 2 bp (trnY-GUA and trnS1-GCU) to 5 bp (trnM-CAU and trnK-CUU) (Figure 4), most of which were 3–4 bp long. The length of the TΨC stem ranged from 3 bp (trnC-GCA and trnV-UAC) to 6 bp (trnL1-UAG) (Figure 4), most of which were 4–5 bp long. There are three types of mismatched base pairs for tRNA: U–U base pairs, A–G base pairs, and non-canonical G–U base pairs (Figure 4). The amino acid accepter stem of trnC-GCA has U–U base pairs (Figure 4), the amino acid accepter stem of trnW-UCA has A–G base pairs (Figure 4), and the anticodon stems of trnW-UCA and trnD-GUC and the TΨC stem of trnS1-GCU have G–U base pairs (Figure 4).

The rrnL and rrnS were 1312 and 786 bp long, respectively. The AU- and GC-skews’ values of rrnL and rrnS were −0.051, 0.305, −0.060, and 0.365, respectively (Supplementary Table S2). The secondary structures of both rrnL and rrnS (Supplementary Figures S1 and S2) contained many helices, similar to those of the *Ha. sinicus* and *Am. aulica* mitogenomes [3,16].

### 3.4. Control Region

**Figure 5:**
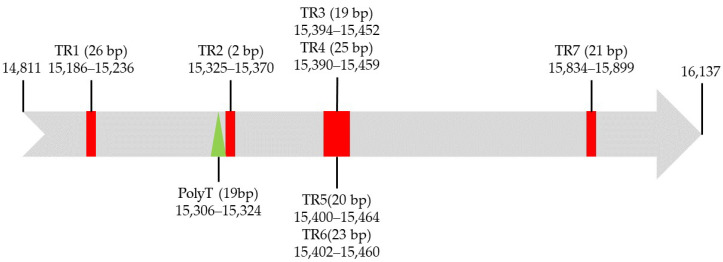
Organization of control region in *G*. *orientalis* mitogenome. The orange block is the tandem repeat region; the green block indicates the poly-T stretch.

The CR, also called the AT-rich region, is 1327 bp in length with an AT content of 88.62% and is located between the rrnS and trnI-GAU genes (Figure 2 and Supplementary Table S2). In the mitogenomes of the subfamily Harpalinae, the AT content of the CR of *G*. *orientalis* was slightly higher than that of *Ha. sinicus*, and lower than that of *Ha. pensylvanicus*. The AT- and GC-skews of CR were 0.005 and −0.125 on the majority strand, respectively, indicating a major-strand compositional bias characterized by a slight excess of A over T nucleotides, and a strong excess of C over G nucleotides. Bias is generally observed in the mitogenomes of members of the subfamily Harpalinae, such as *Ha. pensylvanicus* [17]. The secondary structure of the CR was inferred (Supplementary Figures S3) to contain more than 20 stem-loop structures. A conserved poly-T stretch (19 bp) and seven tandem repeats (TRs) were observed in the CR of the *G*. *orientalis* mitogenome (Figure 5 and Table S3). The total length of the TRs was 238 bp, contributing to 17.94% of the CR size. For TR3-TR6, these four TRs overlapped with each other (Figure 5 and Table S3). The region where TR3-TR6 was located, was the most enriched region of A + T, starting from 15,383 to 15,471, with a total length of 89 bp which was entirely composed of A and T bases; TR2 was a varied and typical microsatellite-like element (TA)24, with a left-flanking conserved polyT stretch (19 bp) (Figure 5).

### 3.5. Phylogenetic Analysis

**Figure 6:**
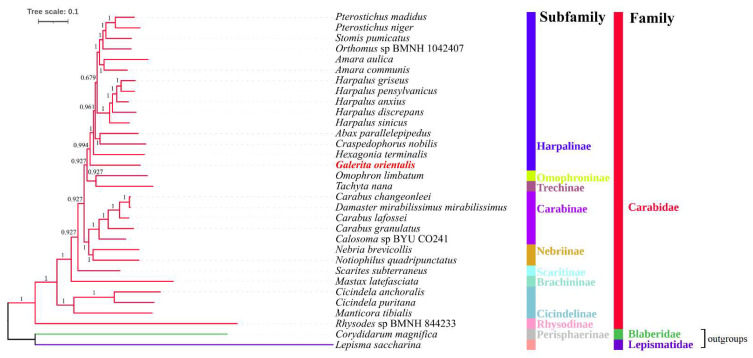
Bayesian-inferred phylogenetic tree of the nucleotide sequences of 13 protein-coding genes (PCGs) of 32 mitogenomes using MrBayes under the GTR + F + I + G4 model. Bayesian inference (BI) poster probability values are indicated near the nodes. The newly determined *Galerita orientalis* is shown in red. The branches of the family Carabidae are shown in red. *L*. *saccharina* and *Co*. *magnifica* are outgroups, and the corresponding branches are shown in green or purple.

**Figure 7:**
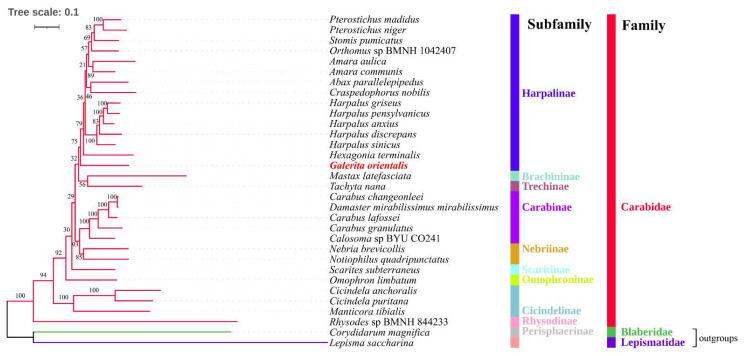
Maximum likelihood phylogenetic tree of nucleotide sequences of 13 protein-coding genes (PCGs) of 32 mitogenomes using IQ tree under the GTR + F + I + G4 model. Maximum likelihood (ML) bootstrap support values are indicated near the nodes. The newly determined *Galerita orientalis* is shown in red. The branches of the family Carabidae are shown in red. *L*. *saccharina* and *Co*. *magnifica* are outgroups, and the corresponding branches are shown in green or purple.

This study’s phylogenetic analyses were based on the nucleotide sequences of the 13 PCGs obtained from 32 mitogenomes (Figure 6 and Figure 7). A total of 11,136 alignment positions were obtained using Gblock [38], from 11,556 alignment positions of the 13 PCGs (Supplementary Table S4). The BI tree provided significantly higher support values than the ML tree for the same dataset, particularly for branches that involved the subfamily Harpalinae relationships of the 13 PCG nucleotide sequences. In the ML tree, significantly low ML bootstrap support values (21 and 36) were observed for the subfamily Harpalinae, which is consistent with the result of a previous phylogenetic study [3]. In the BI and ML trees, nine genera of the subfamily Harpalinae were clustered as (((((((*Pterostichus* + *Stomis*) + *Orthomus*) + *Amara*) + *Harpalus*) + (*Abax* + *Craspedophorus*)) + *Hexagonia*) + *Galerita*) (Figure 6 and Figure 7), which was similar to the results of previous phylogenetic studies [1,3,5,12,16]. At the genus level, *Galerita* is an independent lineage in the topology of the BI and ML trees (Figure 6 and Figure 7). The genus *Galerita* is closely related to the genus Trichognathus in the tribe Galeritini [10,12]. The tribe Galeritini and Dryptini have sister relationships [10]. However, the mitogenomes are unknown. An accurate phylogenetic position within the genus *Galerita* requires additional mitogenome sequences. At the subfamily level of the BI and ML trees, the positions of Brachininae and Omophroninae were unstable. Nevertheless, this study provided a molecular basis for the classification and phylogeny of the family Carabidae, especially the subfamily Harpalinae.

## 4. Conclusions

The genus *Galerita* contains more than 110 species [10], which is relatively disorganized due to simplistic morphological characteristics [9] and the absence of mitogenome molecular phylogenetic evidence. Therefore, we newly assembled the *G*. *orientalis* mitogenome in the present study. Compared to other previously reported mitogenomes of subfamily Harpalinae, all of them presented similar structural characters and nucleotide compositions, which contained 13 PCGs, 22 tRNA, 2 rRNA, and a control region. All 13 protein-coding genes were initiated using a typical ATN (Met) as the start codon, except for *nad1*, which has a TTG as the start codon, and were terminated using a typical TAN stop codon. The 22 tRNA could fold into a typical cloverleaf structure, including trnS1-NCU, which lacks the DHU arm in other mitogenomes of subfamily Harpalinae. Both rrnS and rrnL contained a lot of helices. A conserved poly-T stretch (19 bp) and seven TRs were observed in the CR. The phylogenetic analysis indicated that the genus *Galerita* was an independent lineage. Considering the diversity of the family Carabidae and the limitations of the current mitogenome information, the accurate phylogeny within the genus *Galerita* will require additional mitogenomes.

## Figures and Tables

**Table 1:** The mitogenomic sequences used for phylogenetic analysis in this study.

Family	Subfamily	Species	Whole Length	GenBank Accession Number	Reference
Carabidae	Brachininae	*M*. *latefasciata*	16,735 bp	ON674050.1	Unpublished
	Cicindelinae	*Cicindela anchoralis*	16,388 bp	MG253029.1	[43]
		*Cicindela puritana*	15,676 bp	MW442537.1	Unpublished
		*Manticora tibialis*	16,439 bp	MF497821.1	[44]
	Carabinae	*Carabus changeonleei*	16,831 bp	MG253028.1	[45]
		*Carabus granulatus*	16,918 bp	MN122870.1	Unpublished
		*Carabus lafossei*	16,793 bp	KY992943.1	[46]
		*Calosoma* sp. BYU-CO241	16,462 bp	GU176340.1	[47]
		*Damaster mirabilissimusmirabilissimus*	16,823 bp	GQ344500.1	[48]
	Harpalinae	*Abax parallelepipedus*	17,701 bp	KT876877.1	[15]
		*Amara aulica*	16,646 bp	MN335930.1	[3]
		*Amara communis*	15,745 bp	KX035135.1	Unpublished
		*Craspedophorus nobilis*	15,063 bp	JX412738.1	[49]
		*Galerita orientalis*	16,137 bp	ON920164.1	This study
		*Harpalus anxius*	16,429 bp	ON929899.1	Unpublished
		*Harpalus discrepans*	16,027 bp	OP161482.1	Unpublished
		*Harpalus griseus*	16,972 bp	OP133272.1	Unpublished
		*Harpalus pensylvanicus*	16,434 bp	MN245975.1	[17]
		*Harpalus sinicus*	16,521 bp	MN310888.1	[16]
		*Hexagonia terminalis*	12,639 bp	JX412768.1	[49]
		*Orthomus* sp. BMNH 1042407	16,368 bp	MK692555.1	Unpublished
		*Pterostichus madidus*	18,324 bp	KT876910.1	[15]
		*Pterostichus niger*	17,160 bp	KT876909.1	[15]
		*Stomis pumicatus*	17,265 bp	KT876914.1	[15]
	Nebriinae	*Nebria brevicollis*	21,161 bp	KT876906.1	[15]
		*Notiophilus quadripunctatus*	15,312 bp	MW800883.1	[50]
	Omophroninae	*Omophron limbatum*	15,438 bp	MW800882.1	[50]
	Rhysodinae	*Rhysodes* sp. BMNH-844233	16,315 bp	KX035156.1	Unpublished
	Scaritinae	*Scarites subterraneus*	16,163 bp	OL872182.1	[51]
	Trechinae	*Tachyta nana*	16,165 bp	KX035142.1	[15]
Lepismatidae		*L*. *saccharina*	15,244 bp	MT108230.1	[52]
Blaberidae	Perisphaerinae	*Co*. *magnifica*	16,627 bp	MW630139.1	[53]

**Table 2:** Reads statistics for *G. orientalis*.

Raw Reads Base (bp)	Raw Reads num	Q20 (%)	Q30 (%)	Clean Reads Base (bp)	Clean Reads num	Q20 (%)	Q30 (%)	GC (%)
51,892,104,000	345,947,360	96.90	91.37	43,375,436,400	289,169,576	98.37	93.88	35.88

Abbreviations: Q20, percentage of bases with quality value ≥20; Q30, percentage of bases with quality value ≥30; GC, GC content.

**Table 3:** Organization of *G. orientalis* mitogenome.

Gene	Strand	Location	Size (bp)	Anticodon	Start Codon	Stop Codon	Intergenic Nucleotides
trnI	J	1–65	65	GAT			
trnQ	N	66–140	75	TTG			0
trnM	J	136–206	71	CAT			−5
*nad2*	J	206–1234	1029		ATA	TAA	−1
trnW	J	1232–1306	75	TCA			−3
trnC	N	1333–1396	64	GCA			2
trnY	N	1398–1465	68	GTA			1
*cox1*	J	1458–2997	1540		ATT	T	−8
trnL2	J	2998–3062	65	TAA			0
*cox2*	J	3065–3749	685		ATG	T	2
trnK	J	3750–3820	71	CTT			0
trnD	J	3821–3886	66	GTC			0
*atp8*	J	3887–4048	162		ATC	TAA	0
*atp6*	J	4042–4719	678		ATG	TAA	−7
*cox3*	J	4719–5507	789		ATG	TAA	−1
trnG	J	5509–5576	68	TCC			1
*nad3*	J	5576–5929	354		ATT	TAG	−1
trnA	J	5930–5990	61	TGC			0
trnR	J	5993–6058	66	TCG			2
trnN	J	6059–6124	66	GTT			0
trnS1	J	6125–6191	67	GCT			0
trnE	J	6193–6261	69	TTC			1
trnF	N	6257–6325	69	GAA			−5
*nad5*	N	6324–8052	1729		ATT	T	−2
trnH	N	8053–8117	65	GTG			0
*nad4*	N	8118–9456	1339		ATG	T	0
*nad4l*	N	9450–9743	294		ATT	TAA	−7
trnT	J	9744–9812	69	TGT			0
trnP	N	9811–9875	65	TGG			−2
*nad6*	J	9877–10,401	525		ATT	TAA	1
*cob*	J	10,401–11,537	1137		ATG	TAG	−1
trnS2	J	11,536–11,603	68	TGA			−2
*nad1*	N	11,620–12,570	951		TTG	TAG	16
trnL1	N	12,571–12,636	66	TAG			0
rrnL	N	12,637–13,948	1312				0
trnV	N	13,954–14,024	71	TAC			5
rrnS	N	14,025–14,810	786				0
CR	J	14,811–16,137					

Abbreviations: J, J-strand (the majority strand); N, N-strand (the minority strand).

## Data Availability

The following information was supplied regarding the deposition of DNA sequences: The raw data can be obtained from the Sequence Read Archive at NCBI under accession number SRR20727398. The associated BioProject and Bio-Sample numbers are PRJNA864596 and SAMN30075025, respectively.

## Supplementary Materials

The following supporting information can be downloaded at: www.mdpi.com/article/10.3390/genes13122199/s1, Table S1: Codon number and RSCU of PCGs of *G. orientalis* mitogenome; Table S2: Composition and skewness of genes and CR of *G. orientalis* mitogenome; Table S3: Tandem repeats of CR of *G*. *orientalis* mitogenome; Table S4: Alignment results using Gblocks; Figure S1: Predicted secondary structure of rrnL of *G*. *orientalis* mitogenome; Figure S2: Predicted secondary structure of rrnS of *G. orientalis* mitogenome; Figure S3: Predicted secondary structure of control region of *G. orientalis* mitogenome.

## References

[1] Ober K.A. (2002). Phylogenetic relationships of the carabid subfamily Harpalinae (Coleoptera) based on molecular sequence data. Mol. Phylogenet. Evol..

[2] Bousquet Y., Larochelle A. (1993). Catalogue of the Geadephaga (Coleoptera: Trachypachidae, Rhysodidae, Carabidae including Cicindelini) of America north of Mexico. Mem. Entomol. Soc. Can..

[3] Li Z., Li X., Song N., Tang H., Yin X. (2020). The Mitochondrial Genome of Amara aulica (Coleoptera, Carabidae, Harpalinae) and Insights into the Phylogeny of Ground Beetles. Genes.

[4] Lorenz W. (2005). Systematic List of Extant Ground Beetles of the World.

[5] Ober K.A., Maddison D.R. (2008). Phylogenetic relationships of tribes within Harpalinae (Coleoptera: Carabidae) as inferred from 28S ribosomal DNA and the wingless gene. J. Insect Sci..

[6] Hovorka O. (2012). A new Galerita species from Bolivia (Coleoptera: Carabidae: Galeritini). Stud. Rep. Taxon. Ser..

[7] Hovorka O. (2016). New species of Galerita Fabricius, 1801 from Panama (Coleoptera: Carabidae: Galeritini). Stud. Rep. Taxon. Ser..

[8] Hovorka O. (2017). Three new Galerita Fabricius, 1801 species (Coleoptera: Carabidae: Galeritini). Stud. Rep. Taxon. Ser..

[9] Hovorka O. (2019). Five new species of Galerita from Asia and new distributional records (Coleoptera: Carabidae: Galeritini). Folia Heyrovskyana Ser. A.

[10] Makarov K.V., Matalin A.V. (2021). The preimaginal stages of *Galerita ruficollis* Dejean, 1825 and the position of the tribe Galeritini in the classification of ground beetles (Coleoptera, Carabidae). Zookeys.

[11] Hunting W. (2008). Female reproductive system of the tribe Galeritini (Coleoptera: Carabidae): Structural features and evolution. Ann. Carnegie Mus..

[12] Ober K.A., Heider T.N. (2010). Phylogenetic diversification patterns and divergence times in ground beetles (Coleoptera: Carabidae: Harpalinae). BMC Evol. Biol..

[13] Ober K.A. (2001). The Evolution of Arboreal Carabid Beetles.

[14] Liu Y.-Y., Zhou Z.-C., Chen X.-S. (2020). Characterization of the Complete Mitochondrial Genome of Epicauta impressicornis (Coleoptera: Meloidae) and Its Phylogenetic Implications for the Infraorder Cucujiformia. J. Insect Sci..

[15] Linard B., Arribas P., Andújar C., Crampton-Platt A., Vogler A.P. (2016). Lessons from genome skimming of arthropod-preserving ethanol. Mol. Ecol. Resour..

[16] Yu X., Tan W., Zhang H., Jiang W., Gao H., Wang W., Liu Y., Wang Y., Tian X. (2019). Characterization of the Complete Mitochondrial Genome of Harpalus sinicus and Its Implications for Phylogenetic Analyses. Genes.

[17] Kieran T.J. (2020). Mitochondrial, metagenomic, and phylogenetic analysis of the ground beetle Harpalus pensylvanicus (Coleoptera: Carabidae). Gene.

[18] Bai Y., Ye L., Yang K., Wang H. (2022). Genome Survey and SSR Analysis of *Camellia nitidissima* Chi (Theaceae). Genet. Res..

[19] Bai Y., Gao X., Wang H., Ye L., Zhang X., Huang W., Long X., Yang K., Li G., Luo J. (2022). Comparative mitogenome analysis reveals mitochondrial genome characteristics in eight strains of *Beauveria*. PeerJ.

[20] Chen S., Zhou Y., Chen Y., Gu J. (2018). fastp: An ultra-fast all-in-one FASTQ preprocessor. Bioinformatics.

[21] Dierckxsens N., Mardulyn P., Smits G. (2016). NOVOPlasty: De novo assembly of organelle genomes from whole genome data. Nucleic Acids Res..

[22] Perna N.T., Kocher T.D. (1995). Patterns of nucleotide composition at fourfold degenerate sites of animal mitochondrial genomes. J. Mol. Evol..

[23] Tillich M., Lehwark P., Pellizzer T., Ulbricht-Jones E.S., Fischer A., Bock R., Greiner S. (2017). GeSeq—Versatile and accurate annotation of organelle genomes. Nucleic Acids Res..

[24] Chan P.P., Lowe T.M., Kollmar M. (2019). tRNAscan-SE: Searching for tRNA Genes in Genomic Sequences. Gene Prediction: Methods and Protocols.

[25] Laslett D., Canbäck B. (2007). ARWEN: A program to detect tRNA genes in metazoan mitochondrial nucleotide sequences. Bioinformatics.

[26] Kent W.J. (2002). BLAT—The BLAST-like Alignment Tool. Genome Res..

[27] Stothard P., Wishart D.S. (2004). Circular genome visualization and exploration using CGView. Bioinformatics.

[28] Tamura K., Stecher G., Kumar S. (2021). MEGA11: Molecular Evolutionary Genetics Analysis Version 11. Mol. Biol. Evol..

[29] Kerpedjiev P., Hammer S., Hofacker I.L. (2015). Forna (force-directed RNA): Simple and effective online RNA secondary structure diagrams. Bioinformatics.

[30] Gendron P., Lemieux S., Major F. (2001). Quantitative analysis of nucleic acid three-dimensional structures11Edited by I. Tinoco. J. Mol. Biol..

[31] Lorenz R., Bernhart S.H., Höner zu Siederdissen C., Tafer H., Flamm C., Stadler P.F., Hofacker I.L. (2011). ViennaRNA Package 2.0. Algorithms Mol. Biol..

[32] Gruber A.R., Lorenz R., Bernhart S.H., Neuböck R., Hofacker I.L. (2008). The Vienna RNA Websuite. Nucleic Acids Res..

[33] Mathews D.H., Disney M.D., Childs J.L., Schroeder S.J., Zuker M., Turner D.H. (2004). Incorporating chemical modification constraints into a dynamic programming algorithm for prediction of RNA secondary structure. Proc. Natl. Acad. Sci. USA.

[34] Benson G. (1999). Tandem repeats finder: A program to analyze DNA sequences. Nucleic Acids Res..

[35] Zhang D., Gao F., Jakovlić I., Zou H., Zhang J., Li W.X., Wang G.T. (2020). PhyloSuite: An integrated and scalable desktop platform for streamlined molecular sequence data management and evolutionary phylogenetics studies. Mol. Ecol. Resour..

[36] Katoh K., Standley D.M. (2013). MAFFT Multiple Sequence Alignment Software Version 7: Improvements in Performance and Usability. Mol. Biol. Evol..

[37] Ranwez V., Douzery E.J.P., Cambon C., Chantret N., Delsuc F. (2018). MACSE v2: Toolkit for the Alignment of Coding Sequences Accounting for Frameshifts and Stop Codons. Mol. Biol. Evol..

[38] Talavera G., Castresana J. (2007). Improvement of Phylogenies after Removing Divergent and Ambiguously Aligned Blocks from Protein Sequence Alignments. Syst. Biol..

[39] Kalyaanamoorthy S., Minh B.Q., Wong T.K.F., von Haeseler A., Jermiin L.S. (2017). ModelFinder: Fast model selection for accurate phylogenetic estimates. Nat. Methods.

[40] Ronquist F., Teslenko M., van der Mark P., Ayres D.L., Darling A., Höhna S., Larget B., Liu L., Suchard M.A., Huelsenbeck J.P. (2012). MrBayes 3.2: Efficient Bayesian Phylogenetic Inference and Model Choice Across a Large Model Space. Syst. Biol..

[41] Nguyen L.-T., Schmidt H.A., von Haeseler A., Minh B.Q. (2014). IQ-TREE: A Fast and Effective Stochastic Algorithm for Estimating Maximum-Likelihood Phylogenies. Mol. Biol. Evol..

[42] Letunic I., Bork P. (2021). Interactive Tree Of Life (iTOL) v5: An online tool for phylogenetic tree display and annotation. Nucleic Acids Res..

[43] Wang A.R., Kim M.J., Jeong S.Y., Kim I. (2018). Complete mitochondrial genome sequence of *Cicindela anchoralis* Chevrolat, 1845 (Coleoptera: Carabidae). Mitochondrial DNA Part B.

[44] López-López A., Vogler A.P. (2017). The mitogenome phylogeny of Adephaga (Coleoptera). Mol. Phylogenet. Evol..

[45] Wang A.R., Kim M.J., Hong E.J., Jeong J.-C., Kim S.S., Kim I. (2019). Complete mitochondrial genome sequence of *Acoptolabrus changeonleei* Ishikawa et Kim, 1983 (Coleoptera: Carabidae). Mitochondrial DNA Part B.

[46] Liu N., Wang S., Yang X., Song J., Wu J., Fang J. (2018). The complete mitochondrial genome of *Carabus* (*Damaster*) *lafossei* (Coleoptera: Carabidae). Conserv. Genet. Resour..

[47] Song H., Sheffield N.C., Cameron S.L., Miller K.B., Whiting M.F. (2010). When phylogenetic assumptions are violated: Base compositional heterogeneity and among-site rate variation in beetle mitochondrial phylogenomics. Syst. Entomol..

[48] Wan X., Hong M.Y., Liao A., Kim M.I., Kim K.-G., Han Y.S., Kim I. (2012). Complete mitochondrial genome of a carabid beetle, *Damaster mirabilissimus mirabilissim* (Coleoptera: Carabidae). Entomol. Res..

[49] Timmermans M.J.T.N., Barton C., Haran J., Ahrens D., Culverwell C.L., Ollikainen A., Dodsworth S., Foster P.G., Bocak L., Vogler A.P. (2015). Family-Level Sampling of Mitochondrial Genomes in Coleoptera: Compositional Heterogeneity and Phylogenetics. Genome Biol. Evol..

[50] Raupach M.J., Deister F., Villastrigo A., Balke M. (2022). The complete mitochondrial genomes of Notiophilus quadripunctatus Dejean, 1826 and Omophron limbatum (Fabricius, 1777): New insights into the mitogenome phylogeny of the Carabidae (Insecta, Coleoptera). Insect Syst. Evol..

[51] Kyndt E.C., Kyndt J.A. (2022). Illumina Short-Read Sequencing of the Mitogenomes of Novel Scarites subterraneus Isolates Allows for Taxonomic Refinement of the Genus Scarites Fabricius 1775, within the Carabidae Family. Insects.

[52] Bai Y., Chen J., Li G., Wang H., Luo J., Li C. (2020). Complete mitochondrial genome of the common silverfish *Lepisma saccharina* (Insecta: Zygentoma: Lepismatidae). Mitochondrial DNA Part B.

[53] Bai Y., Yang K., Ye L., Gao X. (2022). Complete mitochondrial genome of *Pseudoglomeris magnifica* (Shelford, 1907) (Insecta: Dictyoptera: Blaberidae). Mitochondrial DNA Part B.

