# Supplementary figures and images for: Complete Mitogenome and Phylogenetic Analyses of Galerita orientalis Schmidt-Goebel, 1846 (Insecta: Coleoptera: Carabidae: Galeritini)
Source: Genes (Basel). 2022 Nov 23;13(12):2199. doi: 10.3390/genes13122199 (PMC9777712; doi:10.3390/genes13122199)

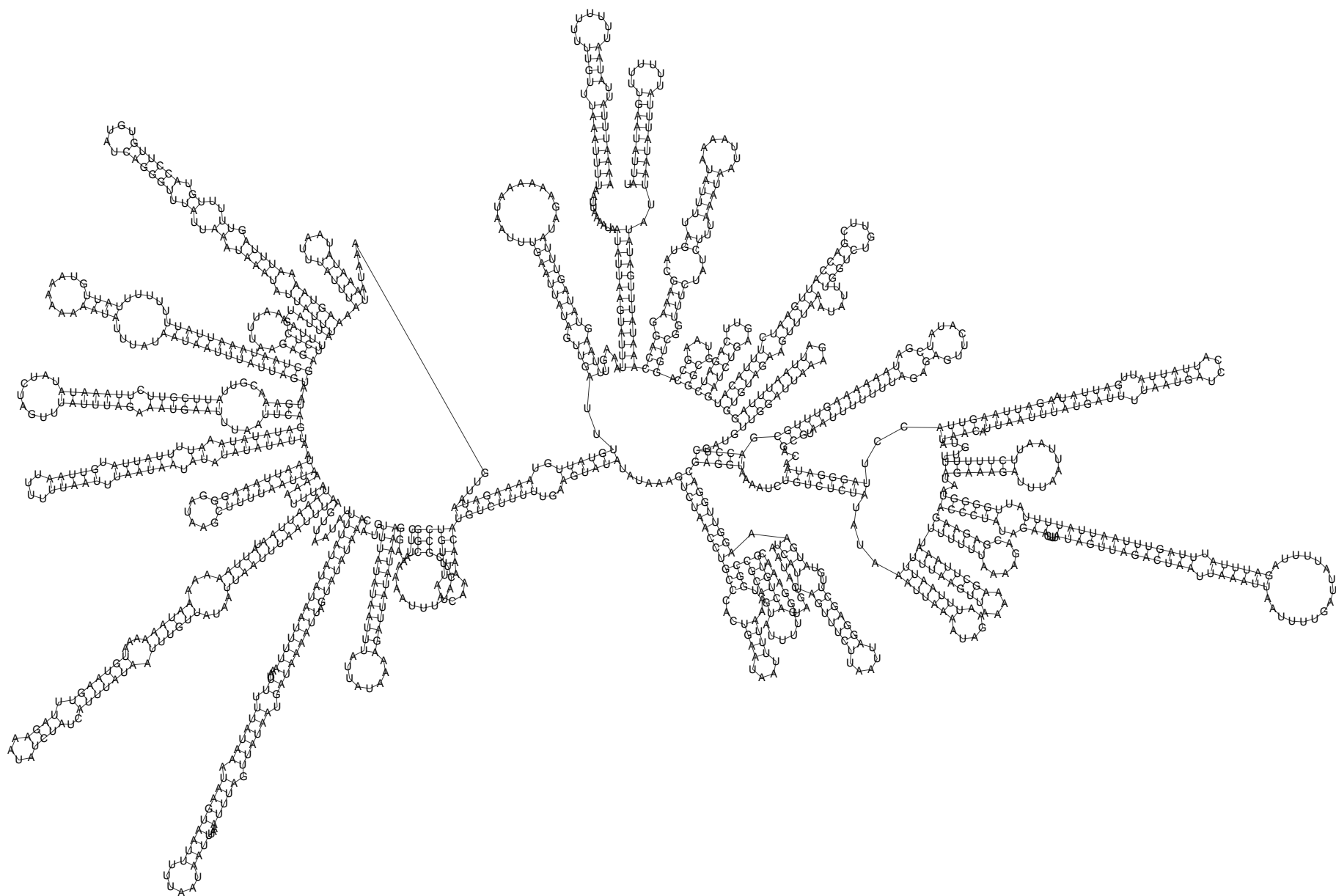

**Figure S1.** Predicted secondary structure of rrnL of *G. orientalis* mitogenome

Supplement: Supplementary file 1 [file genes-13-02199-s001.zip › genes-1980427-supplementary/Figure S1 Predicted secondary structure of rrnL of G. orientalis mitogenome.pdf]

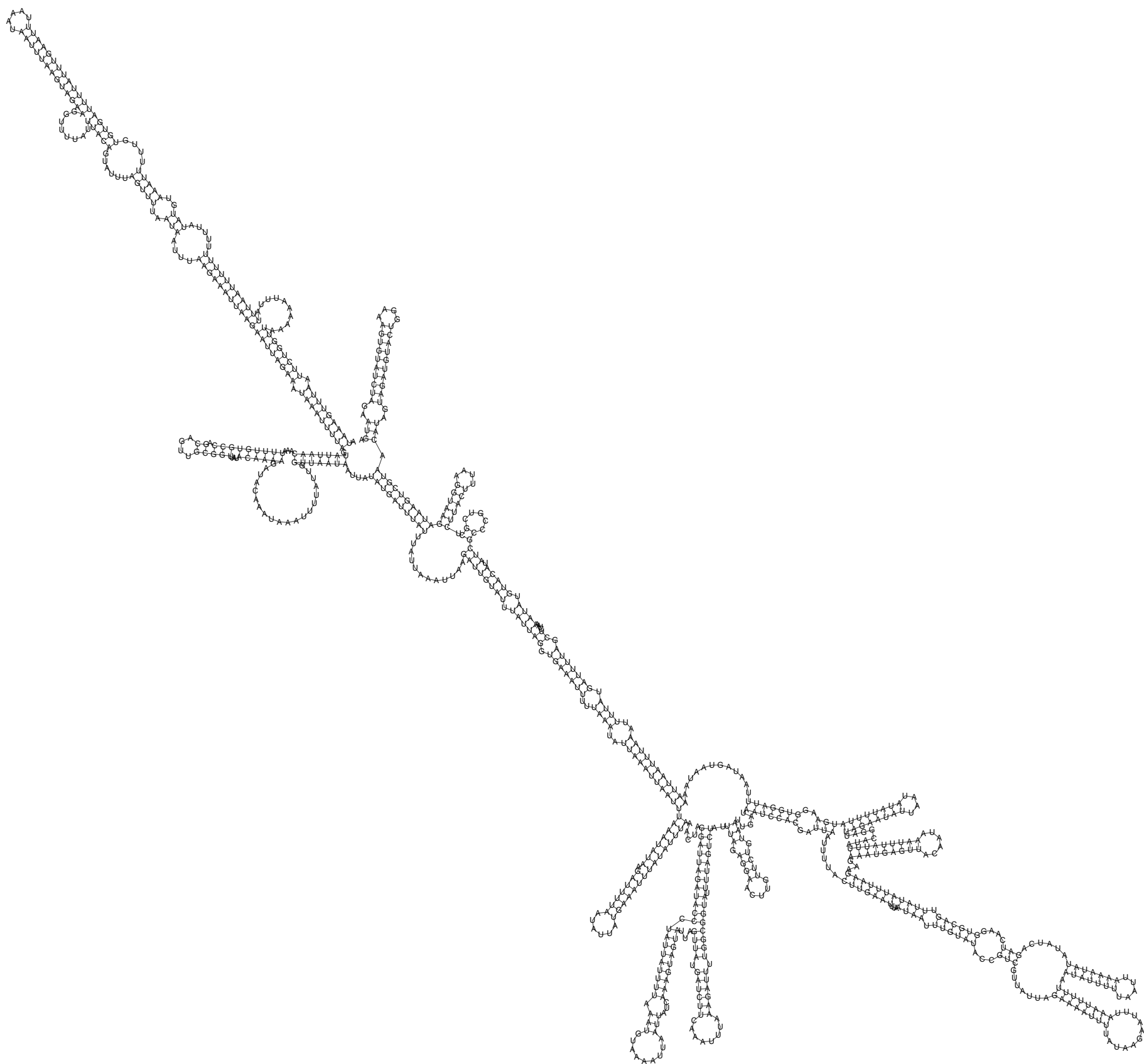

**Figure S2.** Predicted secondary structure of rrnS of *G. orientalis* mitogenome

Supplement: Supplementary file 1 [file genes-13-02199-s001.zip › genes-1980427-supplementary/Figure S2 Predicted secondary structure of rrnS of G. orientalis mitogenome.pdf]

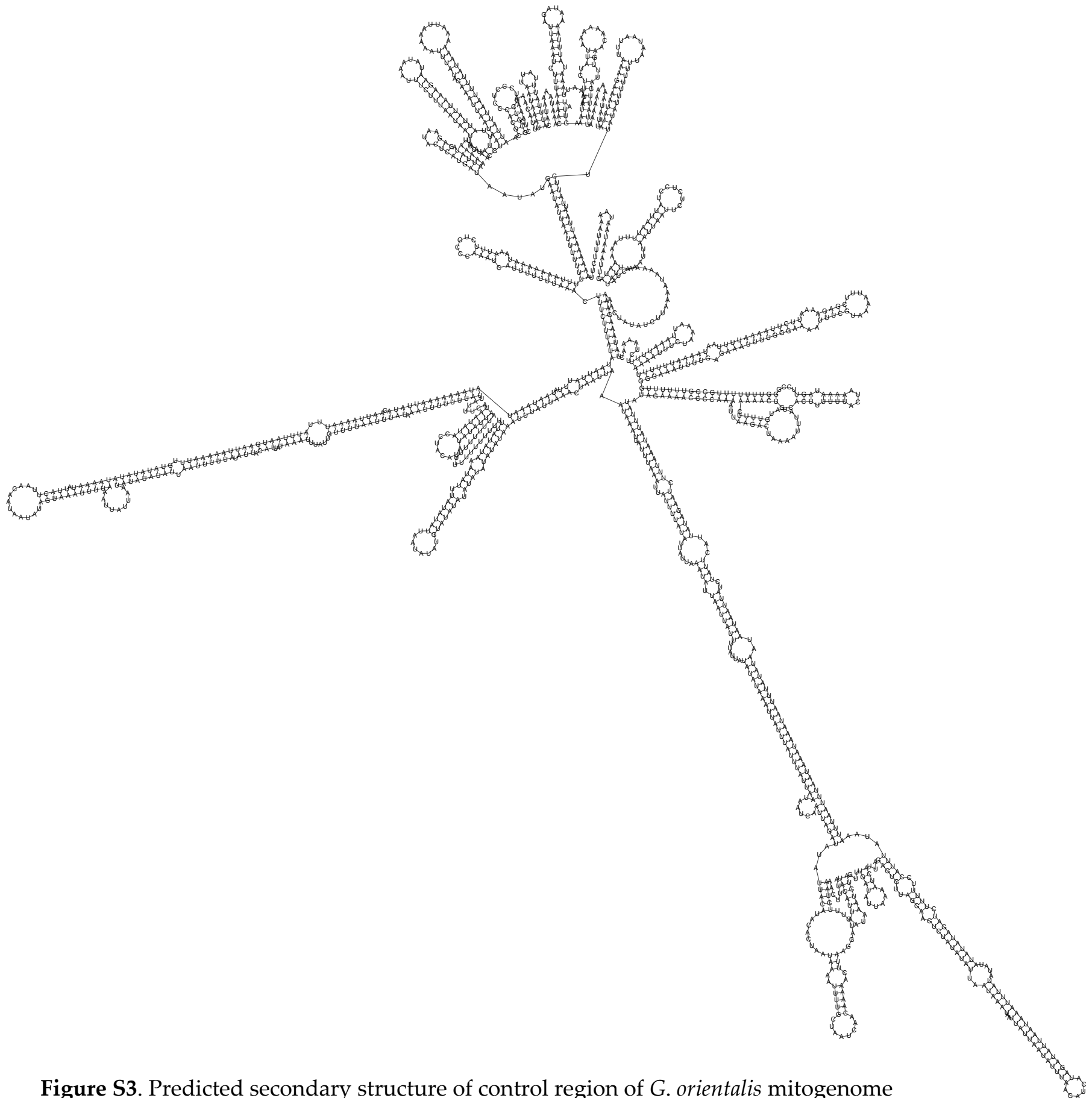

**Figure S3.** Predicted secondary structure of control region of *G. orientalis* mitogenome

Supplement: Supplementary file 1 [file genes-13-02199-s001.zip › genes-1980427-supplementary/Figure S3 Predicted secondary structure of control region of G. orientalis mitogenome.pdf]
